# Starch Granule Size and Morphology of *Arabidopsis thaliana* Starch-Related Mutants Analyzed during Diurnal Rhythm and Development

**DOI:** 10.3390/molecules26195859

**Published:** 2021-09-27

**Authors:** Qingting Liu, Yuan Zhou, Joerg Fettke

**Affiliations:** 1 Biopolymer Analytics, Institute of Biochemistry and Biology, University of Potsdam, Karl-Liebknecht-Str. 24-25 Building 20, 14476 Potsdam-Golm, Germany; qingting.liu@uni-potsdam.de; 2 Max Planck Institute of Molecular Plant Physiology, Am Muehlenberg 1, 14476 Potsdam, Germany; YZhou@mpimp-golm.mpg.de

**Keywords:** starch metabolism, starch granule, starch granule size, starch granule morphology, LCSM, *Arabidopsis thaliana*

## Abstract

Transitory starch plays a central role in the life cycle of plants. Many aspects of this important metabolism remain unknown; however, starch granules provide insight into this persistent metabolic process. Therefore, monitoring alterations in starch granules with high temporal resolution provides one significant avenue to improve understanding. Here, a previously established method that combines LCSM and safranin-O staining for in vivo imaging of transitory starch granules in leaves of *Arabidopsis thaliana* was employed to demonstrate, for the first time, the alterations in starch granule size and morphology that occur both throughout the day and during leaf aging. Several starch-related mutants were included, which revealed differences among the generated granules. In *ptst2* and *sex1-8*, the starch granules in old leaves were much larger than those in young leaves; however, the typical flattened discoid morphology was maintained. In *ss4* and *dpe2/phs1/ss4*, the morphology of starch granules in young leaves was altered, with a more rounded shape observed. With leaf development, the starch granules became spherical exclusively in *dpe2/phs1/ss4*. Thus, the presented data provide new insights to contribute to the understanding of starch granule morphogenesis.

## 1. Introduction

Transitory starch exists as granules in the chloroplasts of autotrophic tissues and organs; its metabolism provides the most fundamental source of carbon and energy for plant growth and development. The starch granule is a highly organized particle with a semicrystalline structure that consists of two biopolymers: amylopectin and amylose [1]. The glucosyl residues in the backbone of amylopectin are linked by α-1,4-linkages and the branching points are interconnected by α-1,6-linkages. Amylopectin accounts for 75–90% of *Arabidopsis thaliana* starches [2]. Amylose has fewer branches than amylopectin does and only represents a small proportion of total starches; however, its precise function is not yet understood [1,3]. Transitory starch is generated throughout the daytime and degraded during the night via strictly coordinated metabolic processes [3].

Over recent decades, starch synthesis and degradation have been extensively studied; however, starch granule initiation remains largely obscure [2,3,4,5]. According to current knowledge, elongating enzymes (e.g., starch synthase, SS) are not able to utilize glucose or ADP-glucose as the starting point for the generation of a glucan chain; thus, starch initiation is believed to rely on a “primer-like” structure formation [4,6]. SS4 was the first enzyme found to play a key role in starch initiation. A lack of SS4 affects the number of starch granules in chloroplasts; thus, only one to two granules per chloroplast in mature and old leaves—and no detectable starch granules in young leaves—were observed under this condition. By contrast, four to seven starch granules per chloroplast were detected in wild-type *Arabidopsis thaliana* [7]. An SS4-interacting protein, Protein Targeting To Starch 2 (PTST2), was reported to bind and transfer suitable oligosaccharides to SS4 [8]. In the corresponding mutant, *ptst2*, a single starch granule per chloroplast was observed, suggesting that PTST2 is also involved in starch initiation. Both *ptst2* and *ss4* revealed a greater accumulation of starch per granule. Interestingly, a lack of SS4 not only changed the number of starch granules but also their shape from the typical flattened discoid morphology to one that was much more spherical. This alteration does not occur in *ptst2*; the granules in *ptst2* were larger but still flattened [7,8].

The plastidial protein Phosphorylase 1 (PHS1), another glucan-forming enzyme, elongates glucan chains that have a degree of polymerization of four (DP4) or greater [4]. Interestingly, the *dpe2phs1* mutant, lacking both PHS1 and Disproportionating Enzyme 2 (DPE2), was also found to have a single starch granule per chloroplast with a more spherical morphology than that seen in *ss4.* However, starch granules were only observed in young leaves and no starch was detected in old leaves [9,10]. DPE2 is important for starch-related maltose metabolism in the cytosol, and its deficiency leads to increased maltose concentration in both the cytosol and the chloroplast [11,12,13]. The amount of SS4 in *dpe2phs1* was not reduced, suggesting an unknown pathway influenced by PHS1 and DPE2 that can affect starch initiation independently of SS4 deficiency [9,10,14]. Surprisingly, an additional lack of SS4 in *dpe2phs1* resulted in aggravated granule morphology, becoming near-perfectly spherical, which implies that SS4, PHS1, and DPE2 work in parallel to influence starch granule morphology via a mechanism that remains to be elucidated.

In addition to the three abovementioned mutants, *sex1-8*, the mutant lacking Glucan Water Dikinase (GWD), has an unusual granule morphology: the starch granules are deformed and thin with irregular edges [15]. GWD uses ATP as a dual phosphate donor, transfers γ-phosphate to water to form orthophosphate, and adds β-phosphate to a glucosyl residue in amylopectin [15,16]. GWD is thought to enable the high-ordered glucan chains at the starch granule surface to become more soluble [17]. The irregular edges of the starch granules in *sex1-8* were concluded to occur as a consequence of uneven metabolism at the starch granule surface. Supportive of this hypothesis, the smaller granules revealed a more even size and morphology than those of the larger ones [15].

The starch granules reflect and integrated various metabolic processes taking place during synthesis and degradation. Thus, understanding the factors that influence starch granule morphology and size allows getting insights into the entire starch metabolism. Further, starch parameters as size and shape are important for the usage of starch, especially for industrial purposes, as adapted parameters are needed for specific applications.

Much of the obtained data pertaining to starch granule morphological parameters were limited to specific time points. Due to analytical limitations, it was impossible to monitor alterations in the granule size and morphology within the diurnal cycle and during leaf development. The analysis of starch granules in their natural environment—without purification—is of significant interest and may lead to a more complete description of the granule parameters.

## 2. Results

### 2.1. In Vivo Imaging by LCSM Can Track and Estimate Diurnal Starch Granule Size and Morphological Alterations

**Figure 1:**
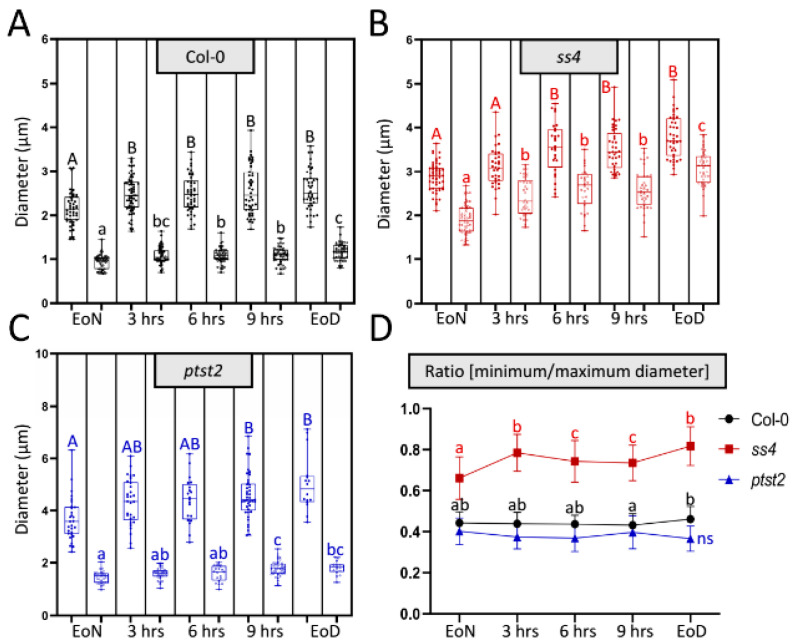
Diurnal alteration on starch granule size and morphology. (**A**–**C**) Changes in maximum (filled dots; first column each) and minimum (empty dots; second column each) diameters of each starch granule in wild type, *ss4*, and *ptst2* during the daytime. Leaf materials were taken from 4-week-old plants. EoN/EoD, end of the night/day; hrs, hours after illumination. (**D**) Change in the ratio of the minimum to the maximum diameter of each starch granule over the day. Mean and SD calculated from *n* > 30. For statistical analyses, one-way ANOVA with Duncan post-hoc test was applied to each group; capital and small letters indicate, respectively, significant differences in maximum and minimum diameters between corresponding groups; *p* < 0.05; n_col-0_ = 43; n*_ss4_* = 28; n*_ptst2_* = 16; ns, no significant difference.

To determine how the abnormal morphologies of starch granules in the various mutants occur, a flexible method that could continuously analyze the size and morphology of starch granules was necessary. However, it was not possible to achieve this via the current widely used methods (scanning and transmission electron microscopy; SEM and TEM, respectively) as they are time-consuming and require many materials and complex sample preparation. Previously, starch granules of the wild type and several starch-related mutants were detected in vivo using laser confocal scanning microscopy (LCSM)-based imaging combined with safranin-O staining. This method was easily and flexibly employed to observe starch granules in different cell types (e.g., mesophyll and guard cells). Starch parameters such as the number of starch granules per chloroplast, size, and morphology were described [18]. Using LCSM, most starch granules in wild type have a lenticular or elliptical shape. The long axis can be regarded as the maximum diameter and the short axis (minimum diameter) can reflect the granule thickness. To test whether this method also allows for the monitoring of diurnal alterations in both granule size and morphology, the diameters of starch granules were measured and maximum and minimum diameters were recorded during the day (Figure 1). The starch granules were not totally degraded at the end of the night; however, the visible granules were fewer at this time than at the end of the light phase. From the end of the night (EoN) to the end of the day (EoD; 12 h light), the maximum diameters of the starch granules in wild type accession Columbia-0 (Col-0) increased from 2.14 ± 0.39 μm to 2.57 ± 0.44 μm (a 20% increase) and the minimum diameters increased from 0.94 ± 0.16 μm to 1.17 ± 0.19 μm (a 24% increase) (Figure 1A). The minimum diameter can reflect the thickness of starch granules that have a typical flattened discoid morphology, as in Col-0 and *ptst2*. The ratio of the minimum to the maximum diameter allows one to estimate alterations in the morphology. In this regard, Figure 1D reveals no changes in the typical morphology of starch granules synthesized in Col-0 throughout the day. Comparably, *ptst2* presented larger starch granules with the typical flattened discoid morphology [8]. Although the maximum and minimum diameters of *ptst2* starch granules increased from 3.68 ± 0.83 μm and 1.45 ± 0.23 μm to 5.03 ± 1.04 μm and 1.80 ± 0.24 μm, respectively (Figure 1C), the ratio remained constant at around 0.4, which was slightly lower than that calculated for Col-0. Nonetheless, the maximum diameter increased more (~1.35 μm, 37%) than the thickness (~0.35 μm, 24%) did, suggesting that the morphology was also maintained in *ptst2* (Figure 1D). The starch granules in *ss4* exhibited a significant difference in morphology compared with those of Col-0 and *ptst2*. It can be clearly seen that both the maximum and minimum diameters increased; the former from 2.88 ± 0.33 μm to 3.80 ± 0.48 μm and the latter from 1.90 ± 0.35 μm to 3.08 ± 0.39 μm (Figure 1B). In contrast to *ptst2* and Col-0, the minimum diameters in *ss4* increased more (~1.18 μm, 62%) than the maximum diameters (~0.92 μm, 32%) did, which implies that the starch accumulated more in the plane of thickness. The ratio of the diameters in *ss4* increased from 0.66 ± 0.1 to 0.82 ± 0.1 (Figure 1D).

### 2.2. In Vivo Status of the Starch Granules in sex1-8 Indicates Alteration of Morphological Features during Leaf Aging

**Figure 2:**
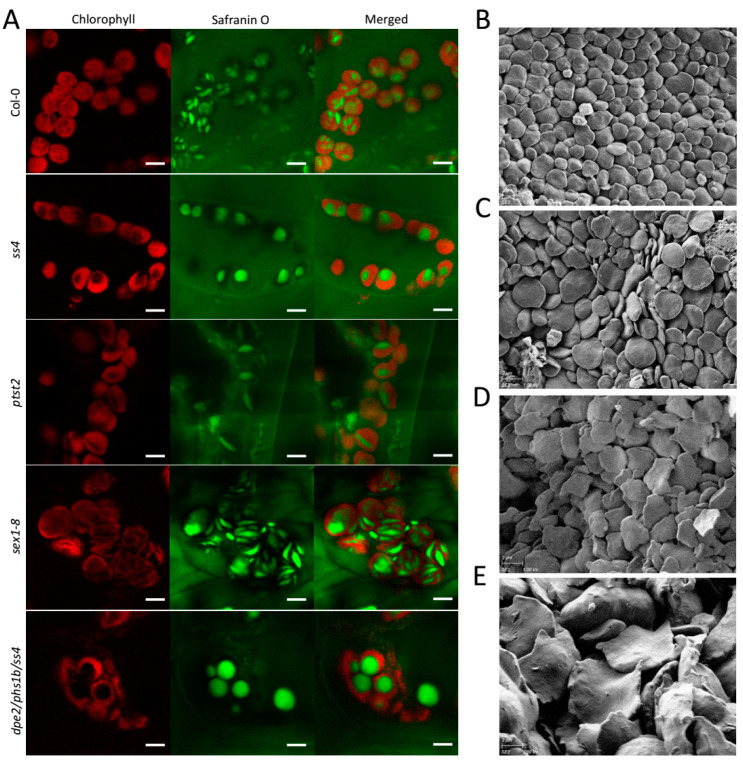
In vivo and in vitro observation of starch granules of *sex1-8*. Starch granules imaged by (**A**) LCSM and (**B**–**E**) SEM: (**A**) Photographs were taken at the end of the day and the scale bar indicated in each picture is 5 μm. (**B**,**C**) In vitro starch granules of the wild type young leaves, Col-0. (**D**,**E**) Starch granules were taken separately from (**D**) young and (**E**) old leaves of *sex1-8*. Magnification of SEM pictures is 5000×. All leaf materials were collected from 4-week-old plants.

For *sex1-8* lacking GWD, starch granules were previously reported to be larger but thin with deformed shape and irregular edges [15]; however, the in vivo situation has not yet been elucidated. An in vivo study of *sex1-8* revealed, as expected, uneven and larger starch granules caused by the near total block of starch degradation [15]. However, the granule morphology was also irregular, with some smaller starch granules presenting the typical flattened discoid morphology and some larger granules that were thicker and more curved (Figure 2A). In contrast, the starch granules of Col-0, *ss4*, *ptst2*, and *dpe2phs1ss4* presented the same morphology as previously reported [8,14,18]. This mixed starch granule morphology might suggest that the abnormal morphology occurred gradually with increasing starch accumulation over time. To confirm the potential morphological alteration in *sex1-8*, the starch granules of young and old leaves were separately extracted and analyzed by SEM. In both young and old leaves, the starch granules were thin with irregular edges; however, in old leaves, the granules were much larger and the morphology was more deformed and curved with more irregular edges than in starch granules isolated from young leaves (Figure 2D,E). The starch granules of young leaves were more flattened, as reported previously [15]; however, the irregular edges of the starch granules in *sex1-8* were not detectable by LCSM. In contrast, the in vitro starch granules from old leaves of Col-0 compared to young leaves showed only an increase in size but no morphological alterations (Figure 2B,C).

### 2.3. Starch Granule Size and Morphological Alterations during Leaf Aging in Various Starch-Related Mutants

**Figure 3:**
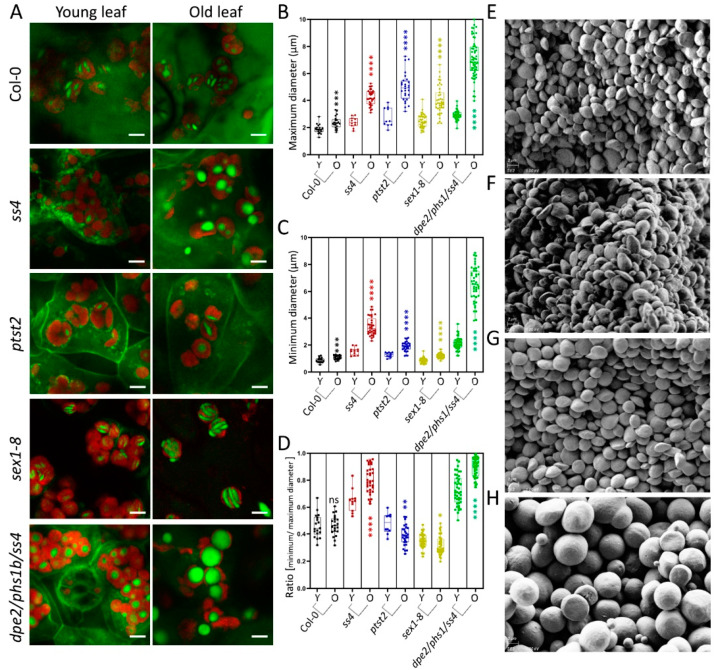
Starch granules in young and old leaves. (**A**) Photographs of 4-week-old plants in the middle of the day; scale bars are 5 μm. (**B**,**C**) The maximum and minimum diameters of starch granules in young (Y) and old (O) leaves of Col-0 and mutants (*n* from 11 to 45; typical measurements are depicted). (**D**) Difference in ratio of starch granule diameter between young and old leaves. (**E**–**F**) Starch granules of young leaves (**E**) and old leaves (**F**) of *ss4*. (**G**–**H**) Starch granules of young leaves (**G**) and old leaves (**H**) of *dpe2/phs1/ss4*. The scale bars in (**E**–**H**) are 2 μm. A student’s *t*-test was performed for each comparison; stars indicate significant differences between young and old leaves of Col-0 and of each mutant. *, *p* < 0.05; **, *p* < 0.01; ***, *p* < 0.001; ****, *p* < 0.0001.

From observation of the starch granules in *sex1-8*, it seems that the morphological features of the granules may be altered with increasing starch accumulation in the mutants during leaf aging. The starch degradation in other tested mutants was also reported to be altered, albeit to a different degree [7,8,14,15,19], which, in turn, could also affect the size and morphology of starch granules. To investigate how starch granules change with starch accumulation throughout leaf aging, the starch granules of young and old leaves were analyzed separately. No macroscopic difference was seen between the starch granules of young and old leaves for Col-0 (Figure 3A). The maximum and minimum diameters were slightly yet significantly increased, but their morphology (ratio) was not changed (Figure 3B–D). Such significant increases were detected in all tested mutants. For *ptst2* and *sex1-8*, the maximum diameters increased by approximately 2.2 μm (81%) and 1.52 μm (62%), respectively (Figure 3B), whereas the minimum diameters only increased by approximately 0.62 μm (33%) and 0.36 μm (42%), respectively (Figure 3C); as a consequence, the thicknesses (ratios) were slightly reduced (Figure 3D). Therefore, the granules were larger but still flattened and discoidal. In the case of *ss4*, only a few starch granules were visible in the chloroplasts of young leaves, as previously reported [7] (Figure 3A). The granules in old leaves were much larger than those in young leaves. The maximum diameters increased by ~1.83 μm (76%), the minimum diameters by ~1.84 μm (119%), and the ratios increased from 0.65 ± 0.08 to 0.80 ± 0.10, suggesting that the granules became more spherical in old leaves than in young ones. *dpe2/phs1/ss4* was reported to present near-perfectly spherical starch granules [14]. This granule morphology was confirmed in both young and old leaves. The maximum and minimum diameters were similarly increased (by ~4.4 μm) between young and old leaves. Interestingly, the ratio of the diameters of starch granules in old leaves of *dpe1/phs1/ss4* increased and the single values were more evenly distributed, compared with young leaves, indicating that the starch granules in old leaves became more spherical and had a more even appearance.

As previously described, the ratio of the maximum to the minimum diameter can reflect the thickness of starch granules. To confirm the morphological alterations, the starch granules of young and old leaves of *ss4* and *dpe2/phs1/ss4* were separately purified and analyzed by SEM. In comparing the starch granules of young and old leaves of *ss4*, the granules appeared larger in old leaves but no morphological difference was observed (Figure 3E,F). The detected change in starch granule size and morphology of *dpe2/phs1/ss4* by LCSM was confirmed by SEM; thus, in young leaves, most starch granules were more rounded than in the wild type, but not yet spherical, whereas nearly all of the starch granules of old leaves were spherical (Figure 3G,H).

## 3. Discussion

Tracking and estimating the size and morphological alterations in starch granules of relevant mutants could provide insight into the underlying molecular mechanisms. Here, we showed for the first time that increases in starch granule size can be monitored during daily starch synthesis for the wild type and some starch-related mutants. Furthermore, we were able to visualize morphology changes during leaf aging. This is the first step toward a deeper analysis of the underlying mechanisms that affect starch morphology. LCSM with safranin-O staining provided the basis for this ability. In our previous studies [18], we demonstrated for the first time that the number of starch granules per chloroplast and the size and shape of these granules were clearly exhibited through the fluorescence of safranin-O. Previously utilized methods, such as SEM or those based on sectioning (e.g., TEM), are strongly limited by intensive material consumption, complicated sample preparation, and fragmentary imaging (due to sectioning). The key advantage of LCSM is the flexible and easy analysis of starch granules in vivo, but it also has clear limitations. For example, the granules in *ss4* and *dpe2phs1ss4* (young leaf) did not demonstrate the typical morphology of Col-0 or *ptst2*, which means that the minimum diameter cannot reflect the exact thickness of starch granules as their orientation cannot be easily determined. Thus, SEM remains the method of choice to determine the exact appearance of starch granules in vitro and to confirm the potential morphological alterations indicated by LCSM.

The in vivo starch granules of *sex1-8* were presented for the first time in this study (Figure 2A) and revealed the differences that exist between young and old leaves. The starch granules of young leaves were thin and flat, whereas the granules in old leaves were more deformed and curved with more irregular edges; this confirms the prior conclusion that smaller granules have a more even appearance than larger ones do [15], not only regarding size but also in terms of morphology (Figure 2D,E). Furthermore, the data clearly indicated that, in *sex1-8*, the ratio of the diameters of starch granules remained nearly constant; thus, the morphology also remained typically thin, flat, and discoidal. One hypothesis to explain the larger and more curved starch granules is that, due to space limitations inside the chloroplast, they are forced to adopt a curved morphology (Figure 2A,E). Starch granules become larger in other mutants, but here a more spherical morphology was always observed (Figure 3). Therefore, although phosphorylation—and thus starch degradation—was severely blocked in *sex1-8*, the morphogenesis of starch granules seemed not to be influenced.

Similar to *sex1-8*, the wild type and *ptst2* revealed flattened starch granules. In contrast, *ss4* and *dpe2/phs1/ss4* presented more spherical granules. The presence of rounded or spherical starch granules seems to rely on a different morphology determination event (Figure 1). In *ss4*, abnormal granule morphology was present in both young and old leaves (Figure 3A) and no obvious morphology changes were detected either during daily starch accumulation or leaf aging (Figure 3E,F). Therefore, we speculate that granule morphology is already determined during the initiation of granules, or that there is no change in the underlying molecular mechanisms, similar to the wild type, *sex1-8*, and *ptst2*.

In *dpe2/phs1/ss4* young leaves, the starch granules were not spherical; however, most granules were more rounded than in the wild type, similar to those seen in *ss4* (Figure 3A,G). In *dpe2/phs1/ss4* old leaves, nearly all starch granules were spherical (Figure 3A,H). This points to a morphological alteration in *dpe2/phs1/ss4* that involves a gradual change from rounded to near-perfectly spherical starch granules. In addition, numerous small spherical starch granules could be found in the old leaves that were not detectable in young leaves. These were much smaller than the granules of young leaves, which implies that they might be new products rather than accumulated starch granules (Figure 3H, Supplemental Figure S2). Taken together, these findings again point to non-identical pathways in *ss4* and *dpe2/phs1* mutants regarding control of starch initiation and its impact on granule morphology.

The data also clearly indicate that alterations in degradation—as, for example, in the case of *sex1-8*—can affect starch granule morphology. In addition, the observed changes during leaf aging (in *sex1-8* and *dpe2/phs1/ss4*) indicate that even potential small alterations in starch synthesis and degradation parameters can, over time, massively influence starch granule morphology, potentially because these small alterations are sustained when starch granules were not totally degraded by the end of the night.

## 4. Materials and Methods

### 4.1. Plant Materials and Growth Conditions

*Arabidopsis thaliana* seeds were sterilized by 70% (*v*/*v*) ethanol and 6% (*v*/*v*) hypochlorous acid with 0.02% (*v*/*v*) Tween 20 and sown on 0.8% (*w*/*v*) agar plates with Murashige and Skoog (MS) medium and no sucrose, pH 5.7. The plates were vernalized in darkness at 4 °C for three days. Seedlings were transplanted to soil after a one-week growing period in an illumination incubator with a light/dark regime (light: 23 °C, 60% humidity, 12 h; dark: 18 °C, 60% humidity, 12 h). Plants were grown in a chamber under the same conditions until sampling. 

The mutants *ptst2* and *sex1-8*, of Col-0 origin, were described previously [15,18]. A T-DNA insertion mutant of SS4 (*SALK_096130*; Col-0) was obtained from the Salk Institute Genomic Analysis Laboratory. Homozygosity was confirmed by PCR (forward primer (*Lp*): *5-CTTCTGGAAGAACAGTTAGAGAAGCTT-3* and reverse primer (*Rp*): *5-GAATGGAGTCCTTTTCCTCCC-3*; T-DNA specific primer *LBb1.3*: *5-ATTTTGCCGATTTCGGAAC-3)*. Genotypical confirmation was performed for *ss4* and *dpe2phs1ss4* as their Col-0 origin was not previously reported (Supplemental Figure S1).

### 4.2. Safranin-O Staining and Laser Confocal Scanning Microscopy

Safranin-O staining and LCSM imaging were performed as described in [18]. To analyze diurnal alterations in the starch granules (Figure 2), leaves of similar age (same verticillate leaf) from 4-week-old plants at the indicated growth time points were used. Pictures were analyzed by ZEN lite 2 software. Single granules were selected and measured with the circle tool according to their maxima and minimal axis. The exact diameter was computed automatically.

### 4.3. Starch Granule Extraction

Leaves of the wild type and mutants were separately collected and classified according to their age as young, mature (not shown), or old leaves when the whole plants were 4 weeks old. The leaf materials were frozen in liquid nitrogen and ground into a fine powder with a mortar and grinding mill. The powder was suspended in 40 mL extraction buffer (20 mM HEPES-KOH, pH 7.4, 0.4 mM EDTA, and 0.05% (*v*/*v*) Triton X-100) and filtered through Miracloth (475855-1R, Millipore Sigma, Darmstadt, Germany) before the filtrate was centrifuged at 4 °C, 1500× *g* for 5 min. Supernatants were discarded and the sediments were resuspended in 40 mL extraction buffer, which was filtered through 100 μm and 30 μm meshes before the filtrate was centrifuged as described above. The resulting sediments were again resuspended in 2 mL extraction buffer and slowly pipetted onto 5 mL Percoll (17-0891-01, GE Healthcare) and centrifuged at 4 °C, 1500× *g* for 15 min. The supernatants were discarded and the sediments/starch granules were washed with water three times and finally dried in a SpeedVac.

### 4.4. Scanning Electronic Microscopy

SEM observation was performed as described previously [15].

### 4.5. Native PAGE and Zymograms

Native PAGE for the detection of DPE2 and PHS1 activity was performed as described in [20].

## Data Availability

Not applicable.

## Supplementary Materials

The following are available online. Figure S1: Genotype confirmation of *ss4* and *dpe2phs1ss4*, Figure S2: In vitro starch granules of *dpe2phs1ss4*.

## Sample Availability

Samples are not available.
