# Supplementary material for: Starch Granule Size and Morphology of Arabidopsis thaliana Starch-Related Mutants Analyzed during Diurnal Rhythm and Development
Source: Molecules. 2021 Sep 27;26(19):5859. doi: 10.3390/molecules26195859 (PMC8510473; doi:10.3390/molecules26195859)
Supplement: Supplementary file 1 [file molecules-26-05859-s001.zip › molecules-1384825-supplementary.pdf]

# Supplementary Figure S1

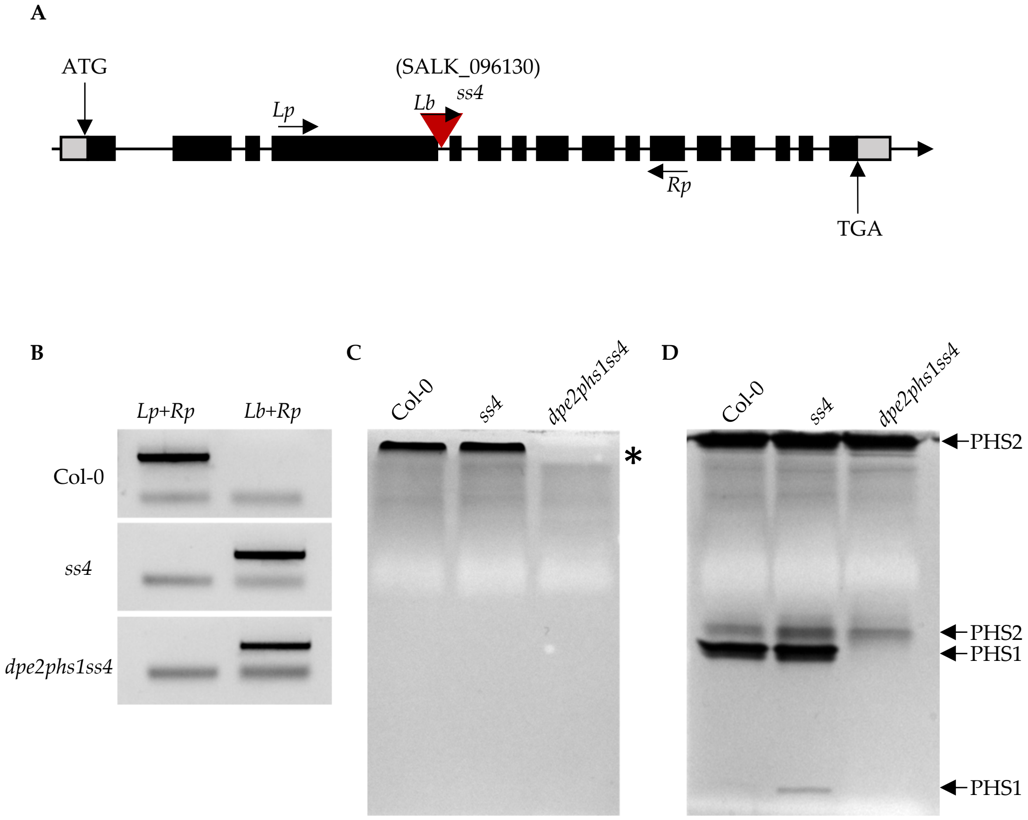


**Figure S1. Genotype confirmation of *ss4* and *dpe2phs1ss4*.** (A) Insertion site as referred to sequence information of TAIR database. (B) Confirmation for the lacking of SS4 in *ss4* and *dpe2phs1ss4*. Primers as indicated in (A). (C) Confirmation of DPE2 activity in Col-0, *ss4* and *dpe2phs1ss4* by Native-PAGE with glycogen. The targeted bands were indicated with star. (D) Confirmation of PHS1 activity by Native-PAGE with glycogen.

# Supplementary Figure S2

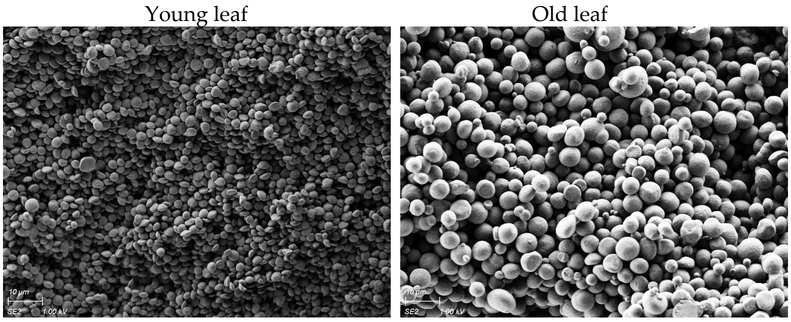


**Figure S2. In vitro starch granules of *dpe2phs1ss4***. The scale bars are 10 μm indicated on each picture.
